# Genetic factors affecting EBV copy number in lymphoblastoid cell lines derived from the 1000 Genome Project samples

**DOI:** 10.1371/journal.pone.0179446

**Published:** 2017-06-27

**Authors:** Rajendra Mandage, Marco Telford, Juan Antonio Rodríguez, Xavier Farré, Hafid Layouni, Urko M. Marigorta, Caitlin Cundiff, Jose Maria Heredia-Genestar, Arcadi Navarro, Gabriel Santpere

**Affiliations:** 1 Institute of Evolutionary Biology (UPF-CSIC), Departament de Ciències Experimentals i la Salut, Universitat Pompeu Fabra, PRBB, Barcelona, Catalonia, Spain; 2 Bioinformatics Studies, ESCI-UPF, Pg. Pujades 1, Barcelona, Spain; 3 Georgia Institute of Technology, Department of Biology, Atlanta, Georgia, United States of America; 4 National Institute for Bioinformatics (INB), PRBB, Barcelona, Catalonia, Spain; 5 Institució Catalana de Recerca i Estudis Avançats (ICREA), PRBB, Barcelona, Catalonia, Spain; 6 Center for Genomic Regulation (CRG), PRBB, Barcelona, Catalonia, Spain; 7 Department of Neuroscience, Yale School of Medicine, New Haven, CT, United States of America; University of British Columbia, CANADA

## Abstract

Epstein-Barr virus (EBV), human herpes virus 4, has been classically associated with infectious mononucleosis, multiple sclerosis and several types of cancers. Many of these diseases show marked geographical differences in prevalence, which points to underlying genetic and/or environmental factors. Those factors may include a different susceptibility to EBV infection and viral copy number among human populations. Since EBV is commonly used to transform B-cells into lymphoblastoid cell lines (LCLs) we hypothesize that differences in EBV copy number among individual LCLs may reflect differential susceptibility to EBV infection. To test this hypothesis, we retrieved whole-genome sequenced EBV-mapping reads from 1,753 LCL samples derived from 19 populations worldwide that were sequenced within the context of the 1000 Genomes Project. An i*n silico* methodology was developed to estimate the number of EBV copy number in LCLs and validated these estimations by real-time PCR. After experimentally confirming that EBV relative copy number remains stable over cell passages, we performed a genome wide association analysis (GWAS) to try detecting genetic variants of the host that may be associated with EBV copy number. Our GWAS has yielded several genomic regions suggestively associated with the number of EBV genomes per cell in LCLs, unraveling promising candidate genes such as CAND1, a known inhibitor of EBV replication. While this GWAS does not unequivocally establish the degree to which genetic makeup of individuals determine viral levels within their derived LCLs, for which a larger sample size will be needed, it potentially highlighted human genes affecting EBV-related processes, which constitute interesting candidates to follow up in the context of EBV related pathologies.

## Introduction

The *Human herpesvirus 4*, also known as Epstein-Barr virus (EBV), belongs to the *gammaherpesvirinae* subfamily and is the causal agent of infectious mononucleosis in humans. It triggers other lymphoproliferative disorders and causes 1% of all cancers, including nasopharyngeal carcinoma (NPC), Hodgkin Lymphoma and Burkitt Lymphoma (BL) [1]. In addition, EBV has also been linked to immune disorders, such as systemic lupus erythematous, multiple sclerosis, and rheumatoid arthritis [2,3].

Many of these EBV-associated diseases display striking differences in prevalence across various regions of the world. For example, BL is most commonly found in Africa, whereas NPC is more prevalent in Asia [4]. While many environmental factors may account for a good proportion of such variation [5], some of the geographical variance in prevalence could be explained by a number of genomic and environmental factors, acting alone or in combination. These include (i) differences in disease risk due to differences in the genetic architecture of the relevant diseases across human populations; (ii) geographic differences in the host’s genetic susceptibilities to EBV infection; (iii) genomic differences between EBV strains across geographical regions; and (iv) pathogenic interactions between variants in the host and the virus genome.

Regarding the genomic variation of the EBV, scarcity of whole-genome data from healthy and diseased individuals with different ethnic backgrounds precludes any *virome-wide* association analysis. However, the recent publication of the first EBV strains from healthy individuals already shows remarkable geographic stratification in the variability of the virus [6]. Genome-wide association studies (GWAS) are commonly used to discover genetic variants contributing to complex diseases and viral infections [7,8]. To date, variation linked to more than 30 human genes has been found associated either with EBV antibody levels or with EBV-related disorders [9]. Interestingly, many of these genes seem to be highly inter-related in the interactome [9]. Moreover, the HLA region in chromosome 6 contains many associations with EBV-related phenotypes. For example, a GWAS study identified multiple, strong associations of EBV anti-EBNA-1 antibody count with genetic factors located in the HLA region [10]. Notably, the same authors found that anti-EBNA-1 antibody levels showed 43% heritability. Anti-EBV antibody levels might not directly reflect individual EBV copy number (number of EBV genome copies per cell) and so it is necessary to ascertain genetic variants of the host directly associated with EBV copy number if any solid inferences are to be made.

A commercial EBV strain (B95-8, derived from a Marmoset cell line) is commonly used to transform B-cells into lymphoblastoid cell lines (LCLs) that, in turn, can be stored for long periods of time in repositories as a source of DNA for large genotypic or genomic studies (*e*.*g*. HapMap or 1000 Genomes Project). These cell lines can be used as a surrogate model to analyse the genetic basis of differences in the copy number of transforming EBV. This approach grounds on the hypothesis that human genetic variants associated with transforming-EBV copy number in their derived LCLs might point to interesting candidate genes to consider in the context of EBV-related diseases. Following this idea, a recent GWAS on EBV copy number conducted on 798 LCLs derived from unrelated HapMap individuals failed to find individual SNPs associated at genome-wide significance levels, despite of 65% of the variance in EBV copy number being explained by all genotyped SNPs [11].

We present the results of a GWAS on an expanded dataset of 1,753 LCLs samples derived from the 1000 Genomes Project (1KGP), where we estimated the number of EBV genomes per LCL using an *in silico* method, which was subject to careful experimental validation. We report several gene candidates and genomic regions linking genetic variants with EBV copy number per LCL in all Populations as well as separately in African, American, Asian and European populations.

## Material and methods

### Samples retrieval from 1000 Genomes Project

The present study involves human genotyping data made publically available by the 1KG project with no need of ethics approval. It also involved LCL from Coriell Institute, to obtain the samples from Coriell we produced the required Statement of Research and Assurance Form for Biomaterials approved by the Institutional Official of the Pompeu Fabra University.

Most of the 1000 Genomes Project samples are coming from lymphoblastoid cell lines (LCLs) maintained at the Coriell Institute for Medical Research. To estimate EBV copy number within these LCLs samples, 1KGP Phase3 aligned reads (release 20130415) were retrieved in low coverage BAM files from 2,535 samples, covering 26 different human populations around the world. We only retained unrelated samples having LCLs as the unique source of DNA (*i*.*e*. excluded samples having blood as DNA source). Annotations provided by the 1KGP were explored to confirm LCLs as the DNA source, which prompted us to exclude a further 367 samples probably having blood as DNA source. Confirmed DNA source information was not available for 179 samples from the ACB, KHV, STU, PUR, and PEL populations, and thus these samples were also excluded from analysis.

### *In silico* EBV copy number estimation

To estimate the number of EBV genome copies per cell (EBV copy number) in a given LCL, we compared the coverage of mapped reads between human genomic regions and the EBV reference genome. To determine EBV coverage, those reads that did not map to the human reference genome (labelled as “unmapped”), or which were already mapped against the EBV reference genome (labelled as NC_007605 in the "mapped" 1KGP alignment files) were retrieved from the 1KGP website. For each LCL sample, we remapped the reads to EBV reference genome (NC_007605) composed of B95-8 strain plus 12Kb of Raji strain to correct the non-natural B95-8 specific deletion. Duplicated paired mappings were removed to avoid PCR duplicates; paired reads not mapping together were filtered out using SAM tools [12]. Only uniquely mapping reads were retained. A total of 2,215 LCL-derived genome samples coming from 4 continents (Europe, Asia, Africa and America), and consisting of 19 populations were selected **(Table 1)** as the final data set for *in silico* EBV copy number estimation. Lastly, we used GATK’s Depth Of Coverage tool [13] to quantify the average EBV coverage per genome sample in a masked version of the EBV reference genome, in which all repetitive and low-complexity regions and the B95-8 specific deletion were excluded (127,219 bp in total). Particular attention was paid to those reads mapping within the B95-8-specific deletion at a median coverage of > = 1 and EBV coverage of < = 1, since they could be an indication of cell lines co-infected with natural EBV strains [6] or of blood as genome source. All such reads were identified and excluded from further analysis.

**Table 1:** 1000 Genomes Project LCL samples used in this study.

1000 Genomes Population Description	1000 Genomes Population Code	1000 Genomes Continent Code	No. Samples used in study
Han Chinese in Bejing, China	CHB	ASN	103
Japanese in Tokyo, Japan	JPT	ASN	101
Southern Han Chinese	CHS	ASN	95
Chinese Dai in Xishuangbanna, China	CDX	ASN	90
Gujarati Indian from Houston, Texas	GIH	ASN	103
Bengali from Bangladesh	BEB	ASN	86
Toscani in Italia	TSI	EUR	104
Utah Residents (CEPH) with Northern and Western European ancestry	CEU	EUR	96
Finnish in Finland	FIN	EUR	96
British in England and Scotland	GBR	EUR	90
Iberian population in Spain	IBS	EUR	106
Yoruba in Ibadan, Nigera	YRI	AFR	103
Luhya in Webuye, Kenya	LWK	AFR	94
Gambian in Western Divisions in The Gambia	GWD	AFR	106
Mende in Sierra Leone	MSL	AFR	74
Esan in Nigera	ESN	AFR	90
Americans of African Ancestry in SW USA	ASW	AFR	59
Mexican Ancestry from Los Angeles USA	MXL	AMR	63
Colombians from Medellin, Colombia	CLM	AMR	94

Next, the hg19 human reference genome was masked to properly estimate the average human genome coverage, excluding regions of copy number variation (CNV), segmental duplication, tandem repeats and repeat masker UCSC tracks. 5 random windows of 1 Kbp size were selected representing "callable" loci of each chromosome and generated a sequence of 110 Kbp size (1 Kbp * 5 windows* 22 chromosomes = 110 Kbp). Reads overlapping these segments were retrieved from the 1KGP website and filtered with the same criteria described above for EBV mappings and the median coverage value was calculated for these regions with the Depth Of Coverage tool.

Finally, EBV copy number was estimated on the basis that the human genome coverage accounts for 2 DNA copies/cell; from this, the number of EBV copies per cell was calculated by the simple procedure of dividing the EBV genome coverage by half of the human genome coverage. Prior to GWAS analysis, and since the range of EBV copy number is very wide and varies among populations, copy number values were normalized by means of inverse rank transformation using GenABEL [14].

### Relative EBV copy number validation by quantitative PCR

DNA was isolated from 13 LCLs samples purchased from Coriell Institute for Medical Research (Camden, USA). Real-time PCR was performed to compare the relative EBV copy number in each LCL. A set of primers and a TaqMan probe were designed to hybridize to EBV-specific region that is repeated 8 times within the virus genome in order to optimize its sensitivity. The amplicon region was selected by breaking the EBV reference sequence in 36 bp fragments, and mapped them against the same reference sequence, in order to evaluate the most repeated regions. Online software tools such as Primer3 [15] and BLAST [16] were used to assist PCR primer design.

Oligonucleotide primer pairs used were Fw: *AAGGGCGCCAGCTTTTCT*, Rv: *ACTTTACAGACAGTGCACAGGAGACT*, and Probe: *FAM-CCCCAGCCTGAGGC-TAMRA*. Real-time PCR was performed on a Quant Studio 12K Flex (Applied Biosystems, Spain) using for each reaction 5μl of TaqMan Universal Master Mix II (Applied Biosystems, Spain), 400–500 nmol of each primer, and 500 nmol of the fluorescent probe. Thermocycler settings were: activation at 50°C for 2 minutes and denaturation at 95°C for 10 minutes, followed by 40 cycles of 95°C 15 seconds denaturation and 60°C 60 seconds annealing/extension.

### Relative EBV copy number stability over time

To determine whether EBV copy number is a stable phenotype over time within LCL, we selected 7 LCLs (1000 Genome Project ID: HG01277, HG00245, HG00362, HG00657, NA18999, NA18502, NA19382). A 1M cells/ml aliquot of each LCL was cultured in 5 ml of fresh RPMI medium (1% Penicillin/Streptomycin, 5% Inactivated fetal serum) until reaching again the concentration of 1M cell/ml. The culture of each LCL was then divided in 3 replicates that were cultured in the same conditions for 6 passages. Each passage was performed when cell reached 1M cells/ml. 1M cells were then transferred to 5 ml of fresh medium and left to grow at 37°C and 5% CO2 (approx. 3–4 days between passages). The spare cells from every passage underwent DNA extraction using E.Z.N.A. Tissue DNA kits (Omega BIO-TEK, Norcross, USA), and subsequently real-time PCR was performed following the conditions stated above. The Neubauer chamber method was applied to count cells. We used an ANOVA test to quantify the relative EBV copy number, using the replicates of every individual as repeated measures for every single passage.

### Genotype data

We retrieved the genotype information from the LCL samples under study in VCF format from the 1KGP (Phase 3) website. The file for the whole project contained around 39 million variants. A total of 5 subsets of samples were prepared on continent basis: (1) All Populations subset, including individuals from all populations and from all continents together, and four continent-wise subsets, namely (2) Europeans, (3) Asians, (4) Africans and (5) Americans. We excluded from each subset all variants with MAF<5% and SNPs falling in regions of the genome containing CNVs identified in the context of the 1KGP or in UCSC tracks of segmental duplications, repetitive and low-complexity regions. Finally, PLINK was used to test Hardy-Weinberg equilibrium (HWE) failures and SNPs with HWE test p-values < = 0.01 were discarded. Final SNP subsets included from ~880k to ~3.5M markers.

### GWAS analysis

We used a linear mixed model as implemented in GEMMA software which fits a linear mixed model (LMM) for SNP-phenotype association testing accounting for population stratification and structure [17]. We calculated the inflation factor (λ) and generated quantile-quantile (qq) plots to compare genome-wide distribution of p-values produced by the association analysis. Inflation factor (λ) values ranged from 0.94 to 1 in all subsets analyzed **(S1 Fig)**, confirming no major inflation of false positives due to unaccounted population substructure.

### Region based SNP analysis

Isolated GWAS hits without clear LD patterns with nearby SNPs might represent spurious associations with EBV copy number. To ascertain biological signals from noise, we selected all GWAS SNPs with p-values <10^−5^ and clumped them into 200 Kbp windows having at least 2 SNPs in each clump and showing among them an LD r^2^ of at least 0.8. We intersected these genomic regions across different populations to check for inter-continental replicability. This approach has the additional advantage of being useful to identify regions associated to EBV copy number that are common across populations even in absence of genome-wide significant SNPs.

### VEGAS analysis

We used VEGAS2 [18] to perform a gene-based analysis of the results from GWAS studies. VEGAS2 incorporates evidence for association from all SNPs across a gene and accounts for gene size (number of SNPs) and linkage disequilibrium (LD) between SNPs by using simulations from the multivariate normal distribution. The 1KGP populations were used as the reference data for pairwise LD correlations. SNPs were allocated to one or more autosomal genes using gene boundaries of ± 50 Kbp [19]. All ranked gene lists produced by VEGAS2 were then used to identify over-represented enriched GO terms using the online tool Gorilla [20].

## Results

### *In silico* EBV copy number estimation

**Fig 1:**
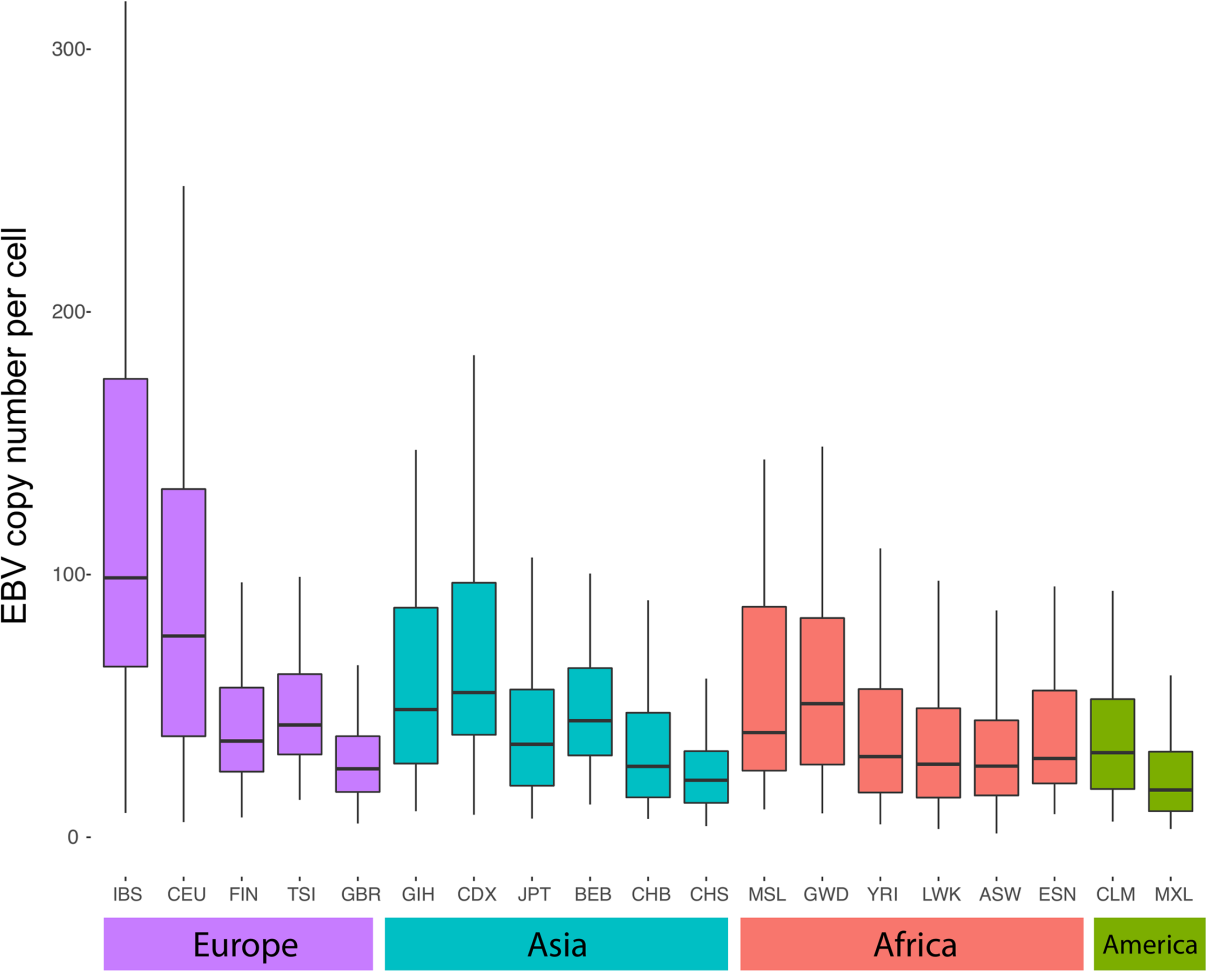
Distribution of EBV copy number across LCL samples. Boxplot showing the distribution of EBV copy number in LCLs across 1000 Genome Project populations. (Population acronyms are explained in Table 1).

We estimated EBV copy number in a total of 2,215 LCL genome samples from the 1KGP by comparing the EBV coverage against the human genomic coverage. All samples having a mean EBV coverage of less than 1 and having coverage in the B95-8 specific deletion were excluded, the latter being indicative of the presence of natural EBV [6]. A total of 1,753 LCL samples were retained for GWAS analysis **(S1 Table)**. EBV copy number ranged from 2 to 500 copies/cell and showed significant differences at the level of populations and continents (ANOVA p-value <2e-16) **(S2 Table)** For instance, the overall EBV copy number in Europeans was significantly higher than the rest of populations and continents. Within Europeans; EBV copy number were higher in IBS and CEU than in FIN, GBR and TSI populations **(Fig 1).** We observed no difference between male and female LCL samples.

### EBV copy number validation by qPCR

**Fig 2:**
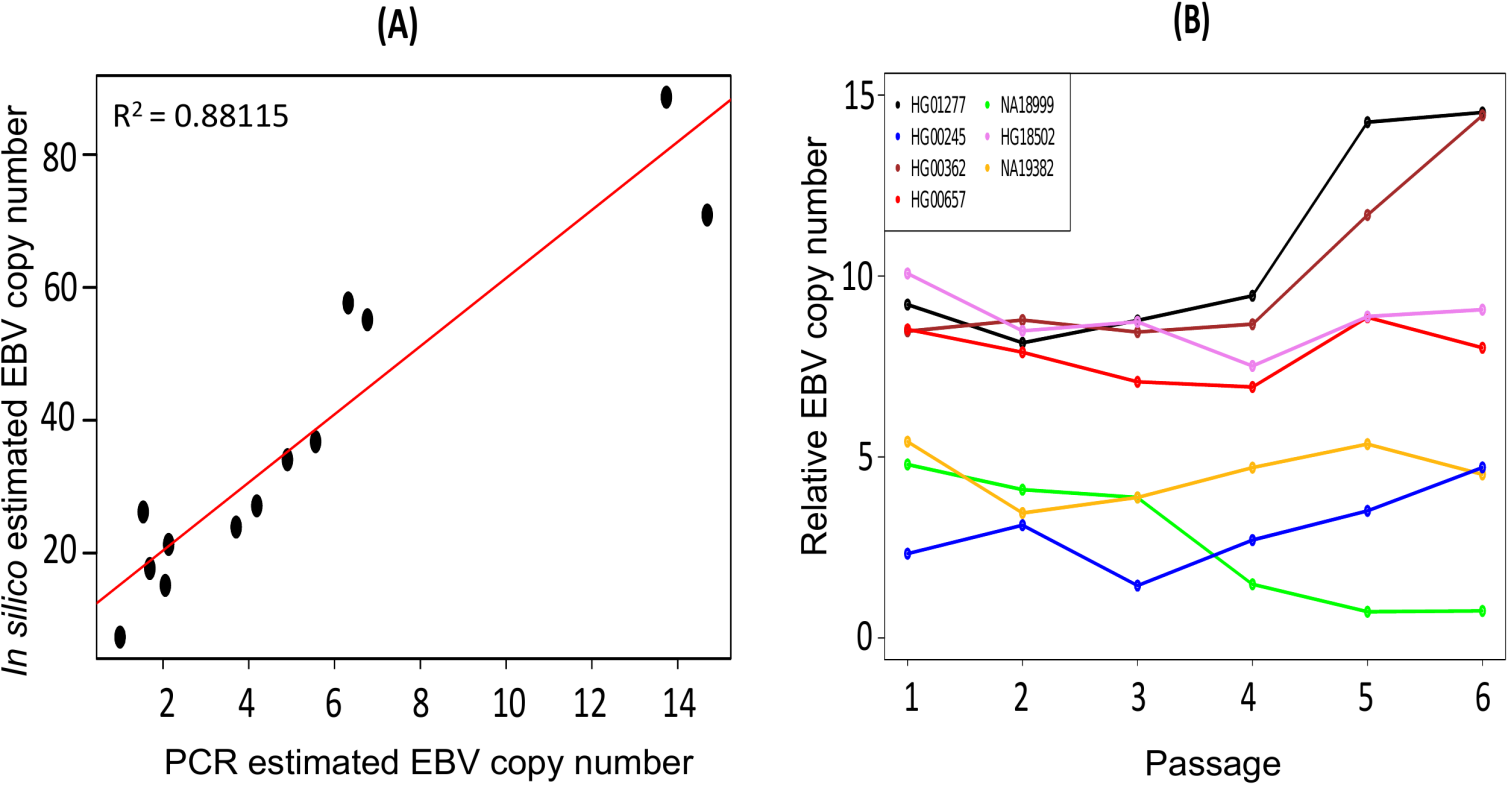
Validation of *in silico* EBV copy number estimation and EBV stability over time. Correlation between EBV copy number for 13 LCL samples as determined by real-time PCR (X-axis) and *in-silico* (Y-axis) **(A)**. Relative real-time PCR measurements of EBV copy number in 7 LCLs (shown with different colors) cultured for 6 passages. Viral copy number tends to be stable within one LCL when compared to inter-strain variation **(B)**.

EBV copy number was quantified by real-time PCR on 13 LCLs samples derived from 1KGP individuals for which the viral copy number had also been estimated by our *in silico* approach, covering a representative range of EBV copy number values. The comparison between our *in silico* and real-time PCR quantifications showed a high correlation (r^2^ = 0.88, p = 0.00007) **(Fig 2)**, which supports and validates the *in silico* EBV copy number estimation approach. In addition, we note that the two estimates were performed on different aliquots from the same individual, which suggests high stability of the viral content within one sample.

### EBV copy number stability over time

In order to specifically interrogate the EBV copy number stability along cell passages, we cultured 7 EBV-transformed LCLs and collected cells at 6 different time points. The experimental design equated to a two-way factorial analysis of variance (ANOVA) with LCL as fixed effects and passages as a random factor nested in LCL lines. The analysis allowed identifying quantitative differences in EBV copy number stability. Result showed that relative EBV quantification by real-time PCR is statistically different between different LCLs and passages (**S3 Table).** This analysis further indicated that 18% of the variation (R^2^) was explained by the passage factor, compared to the 72% explained by inter-individual variation, with a 4-fold higher effect of the latter **(S3 Table).** Although significant, the overall analysis showed that variation during the passages within a LCL is substantially lower than variation between them. This confirmed that EBV copy number is a stable phenotype especially in the context of inter-individual variation **(Fig 2)**.

### SNP-based association test

We retrieved SNP data from the 1KGP for the 1,730 LCLs samples included in this study. We applied several filters to these SNPs (see methods) including CNV removal, MAF<5%, and Hardy-Weinberg equilibrium test, which left us with a total of 0.88, 3.5, 2.7, 2.1, and 2.5 million SNPs in each subset; respectively All populations, African, American, Asian and European populations subset. These SNPs were tested for association with EBV copy number from the All Populations, Asian, African, American and European population subsets, individually. To control for global differences in viral copy number among populations due to unaccounted covariates (i.e. CEU LCLs are older than those from other populations [21]) we rank-transformed our estimated population-wise. The distribution of observed p-value was generally slightly lower than the expected distribution (the estimated inflation factor (λ)), indicating no systematic increase in false-positive hits as a result of population stratification.

Despite of suggestive p-values in the range of *P*~10^−7^ to *P*~10^−6^ in all the studies, **(S4 Table)**, we detected a single signal significant at genome-wide Bonferroni significant levels in the GWAS with African samples (rs6105452, near the *MACROD2* gene, with a p-value of 1.97E-08). For this and other top GWAS-SNP, we investigated its LD pattern by measuring r^2^ values between top SNP and surrounding variants using Locus zoom [22]. We examined rs6105452 SNP in LocusZoom using the African LD map of 1KGP populations. The analysis showed a small peak crowned by rs6105452 with no surrounding highly significant EBV copy number associated variation **(S2 Fig)**; and therefore, it was difficult to determine whether this represented a spurious signal. We decided to focus only on those signals with support of the genomic context (see below).

### Region based SNP association detection and annotation

**Fig 3:**
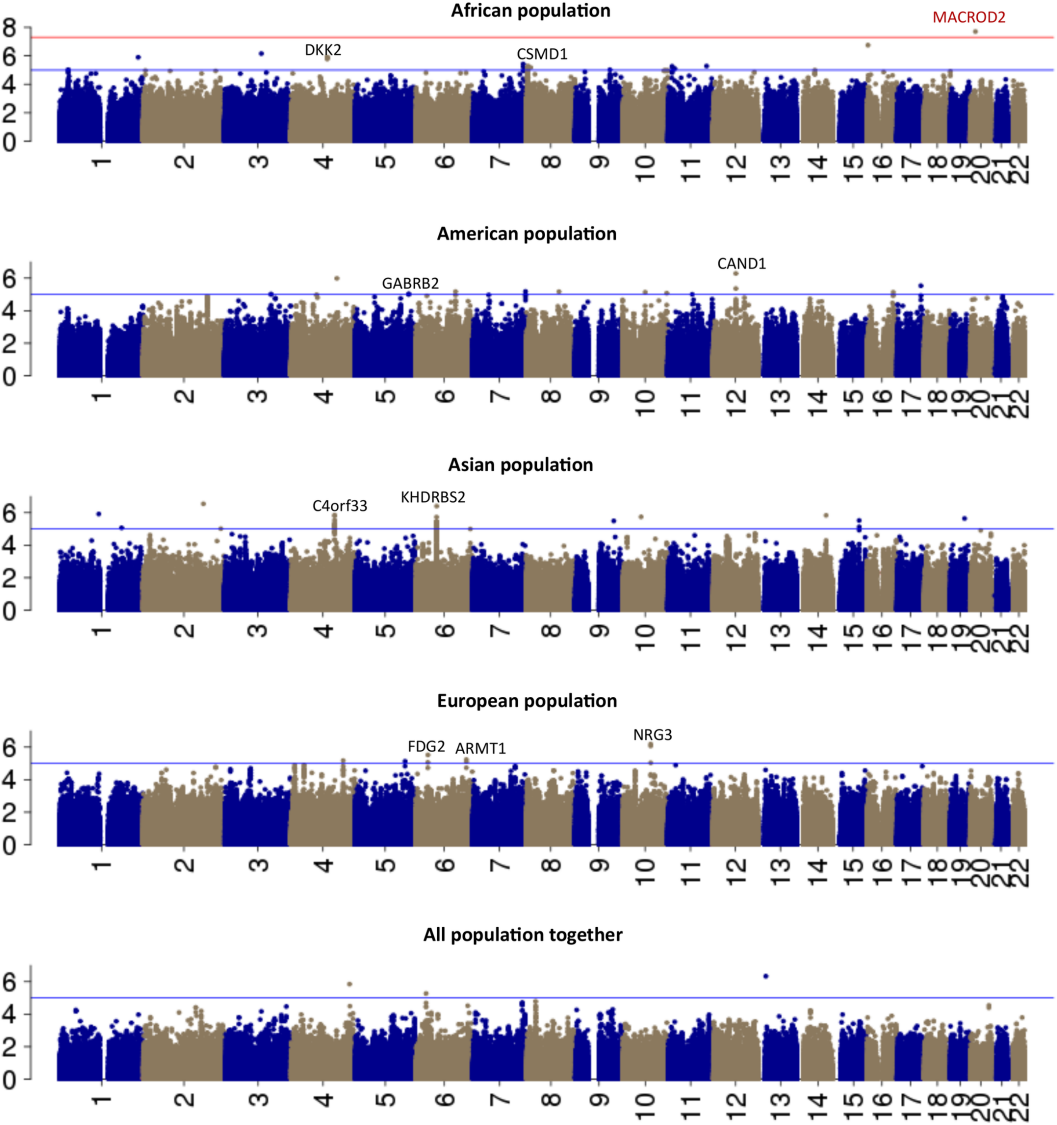
GWAS results Manhattan plot. Manhattan plots for Asian, African American and European population subsets showing top hits from each continent. The blue line indicates p-value of 10^−5^ and red line indicates p-value of 10^−8^.

**Fig 4:**
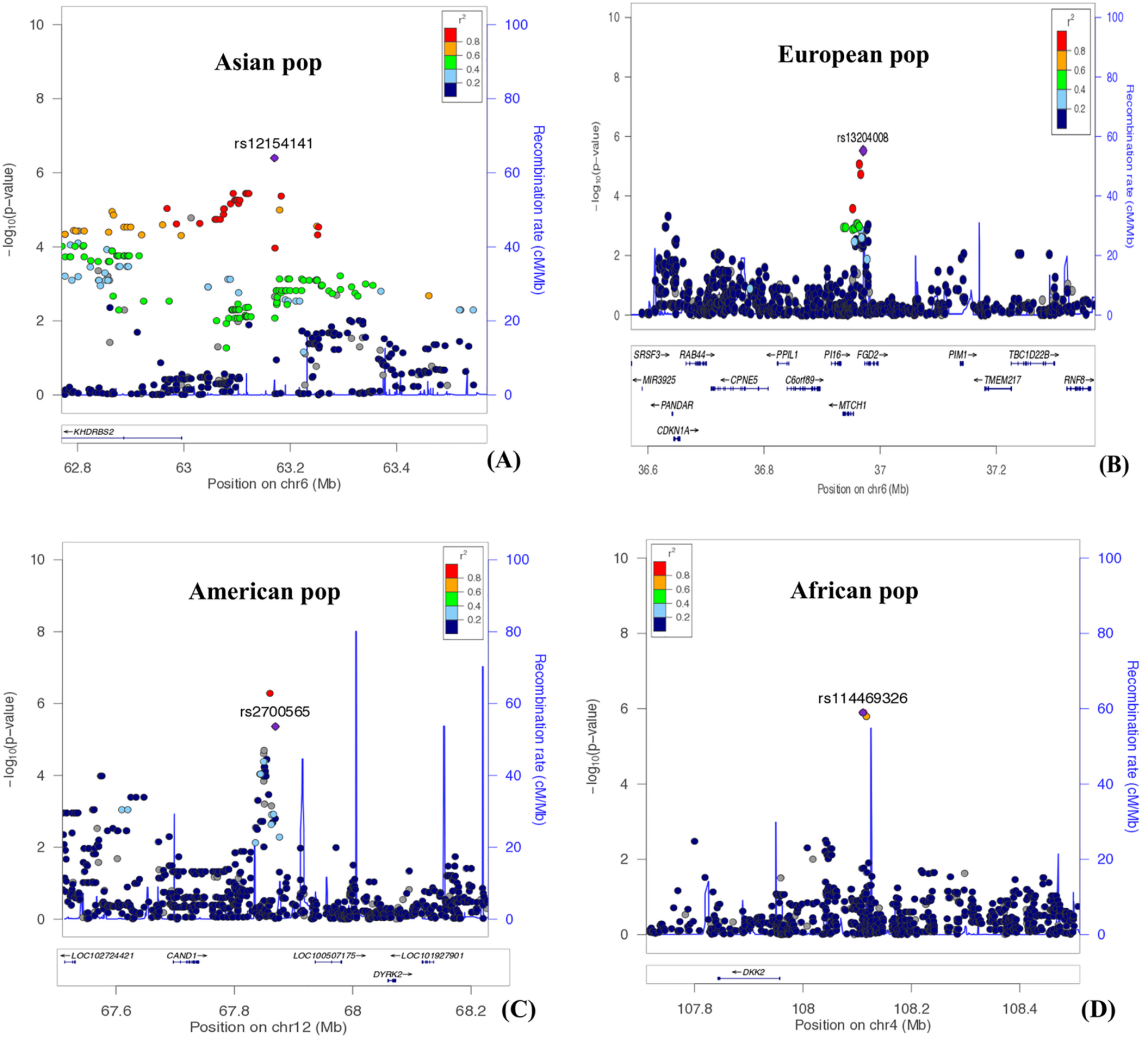
Regional association plots. Regional association plot for Asian **(A)**, European **(B)**, American **(C)** and African population **(D)** subsets produced by Locuszoom showing top SNPs from each population subset (in purple) and surrounding SNPs in the region colored by LD (r^2^) with the top SNP. Lower panel shows genes annotated within this region. Solid blue lines represent recombination rates.

To discriminate loci significantly associated with EBV copy number from noise we generated regions of the genome containing at least 2 SNPs in linkage disequilibrium (r^2^>0.8) with p-values <10^−5^, which we labeled as significant clump regions. We identified 2, 3, 4, and 3 candidate genomic regions in the Asian, European, American and African subsets, respectively (**Table 2**) **(Fig 3)**, whereas no loci in All Populations subset satisfied criteria of a significant region. None of the regions identified was shared among populations. Here, we report study-by-study details on the annotation of significant regions containing clumped SNPs with a p-value < 10^−5^ **(Table 2) (Fig 4)**.

**Table 2:** Top EBV copy number-associated regions in the different population subsets.

Clump Id	Chr	Region_start position	Region_end position	No. of SNPs (p<10^−5)^	No. of SNPs in clump	Top SNP (rs id)	Top SNP (p-value)	SNP feature	Gene name and distance from gene in bp
**Asian Population subset**									
C1	Chr6	62886732	63252602	17	36	rs12154141	4.01E-07	intergenic	KHDRBS2(dist = 173973)
C2	Chr4	130232478	130277236	15	27	rs5861895	1.43E-06	intergenic	C4orf33(dist = 231540)
**European Population subset**									
C1	Chr6	36952226	36970610	2	4	rs13204008	3.02E-06	intergenic	MTCH1(dist = 16283),FGD2(dist = 2813)
C2	Chr6	151791737	151800630	2	4	rs367916962	7.82E-06	intergenic	ARMT1(dist = 5010)
C3	Chr10	84386093	84387269	2	2	rs671631	9.26E-06	intronic	NRG3
**American Population subset**									
C1	Chr3	138353555	138562535	2	19	rs388649	9.89E-06	intergenic	PIK3CB(dist = 18608)
C3	Chr5	160519438	160521734	3	3	rs1387611	6.64E-06	intergenic	GABRB2(dist = 195873)
C4	Chr12	67847460	67857853	2	2	rs2700565	4.35E-06	intergenic	CAND1(dist = 149381)
**African Population subset**									
C1	Chr4	108110200	108111569	4	4	rs114469326	1.27E-06	intergenic	DKK2 (dist = 153436)
C2	Chr7	150025367	150027324	2	3	rs11764936	5.88E-06	intronic	LRRC61
C3	Chr8	5152925	5162760	7	43	rs10099002	5.07E-06	intergenic	CSMD1(dist = 310432)

The top EBV copy number associated region in the Asian subset corresponds to *Chr6*:*62886732–63252602*, centered on rs12154141 (p-value 4.01E-07) that locates in an intergenic region upstream to the *KHDRBS2* gene (dist = 174 Kbp). *KHDRBS2* encodes an RNA binding protein involved in regulating alternative splicing. It could function as an adaptor protein during mitosis and it has been reported to interact with the product of the EBV early gene BSLF2/BMLF1 [23]. The other significant locus in Asians spans the *Chr4*:*130232478–130277236* region, including rs5861895 present in the intergenic region close to C4orf33 (chromosome 4 open reading frame 33). Although no functional information is available for this protein, it has been identified as a multiple sclerosis susceptibility gene [24].

The top significant region in Europeans, *Chr6*:*36952226–36970610*, includes the SNP rs13204008 (p-value, 3.02E-06), near *FGD2*. This gene plays a key role in GPCR and Rho-GTPases signaling pathways. Antigen presenting cells such as B-lymphocytes express FGD2 [25] and, importantly, its paralog *FGD4* has been implicated in LMP1 activation of *CDC42* [26].

Other significant regions in Europeans were close to genes *NRG3* and *ARMT1*. *NRG3* has been shown to trigger activity of the tyrosine phosphorylation of ERBB4, which ultimately influence many cellular processes such as proliferation, migration and differentiation. This gene represents a susceptibility locus at Chr10q for schizophrenia [27,28]. *ARMT1* encodes a protein involved in DNA damage and has been identified as a potential target in breast cancer [29].

Americans showed a top associated locus in *Chr12*:*67847460–67857853* region, containing the SNP rs2700565 (p-value, 4.35E-06) ~1.4 Kbp from *CAND1* gene. *CAND1* is a one of the member of ubiquitin ligases involving in regulation of cell cycle, signal transduction and transcription processes [30,31]. An analysis of 13 prostate cancers showed that overexpression of *CAND1* resulted in malignant progression [32]. Work by Gastaldello et al. [33,34] showed a relationship between *CAND1* and the EBV-encoded deubiquitinating and deneddylating enzyme BPLF1. This tegument protein binds to cullins to prevent the recruitment of *CAND1* to the deneddylated cullin-RING ubiquitin ligases (CRLs) [33].

The two other significant regions in Americans point at genes *GABRB2* and *PIK3CB*. GABRB2 gene encodes a multi-subunit chloride channel receptor involved in neurotransmission in the central nervous system. *PIK3CB* is a lipid kinase involved in many cell functions including the activation of neutrophils.

The top significant cluster in Africans maps in *Chr4*:*108110200–108111569*, close to the *DKK2* gene. *DKK2* encodes an inhibitor of the Wnt/beta-catenin signaling [35], whose dysregulation may result in tumorigenesis. *DKK2* epigenetic modification also plays an essential role in Wnt/β-Catenin signaling [36–38]. The two other significant regions in Africans pointed at *CSMD1* and *LRRC61*. *CSMD1* encodes a candidate tumor suppressor gene abundantly expressed in neuronal cells and epithelial cells [39]. A GWAS study suggested association of this gene with multiple sclerosis [40]. *LRRC61* contains no annotated functions.

### Gene-based association test (VEGAS)

We applied VEGAS2 to obtain gene-based measures of association with EBV copy number and obtained lists of genes ranked by p-value **(S5 Table)**. None of the genes in any subset survived the filtering by false discovery rate, and thus we do not report any particular genes. Rather, we investigated the enrichment in particular GO terms of top ranking genes in each population using GORILLA, and online tool which searches for GO enrichments in ranked lists. After correction for multiple testing, *homophilic cell adhesion via plasma membrane adhesion molecules* were observed in American (FDR q-value = 0.074) and European (FDR q-value = 0.069) populations. In addition, *cell-cell adhesion via plasma-membrane adhesion molecules* (FDR q-value = 0.023) was found enriched in European populations. Looking closely to the specific genes triggering those enrichments we detected a large group of proto-cadherines, clustered in the genome, that constitute the major proportions of genes in the *hemophillic cell–adhesion* category in Americans (19 of the 20 genes). Adhesion categories in Europeans also included many proto-cadherines but also many other cell-adhesion-related genes. No GO term showed a significant enrichment in Asian, African and All populations subsets.

### GCTA analysis

We aimed to estimate the proportion of heritability explained by the whole set of genotyped SNPs used in this GWAS. To that effect we used the GCTA tool [41] and in order to account for population structure, we considered as covariates the first ten dimensions of a multidimensional scaling of the identity-by-state matrix. Using untransformed EBV copy number measures we obtained a proportion of variance in All Population subset explained by the analyzed SNPs of 0.78 (n = 1730, SE ± 0.16, P = 9.076e-07). This result was apparently consistent, given the confidence intervals, with the 0.65 of variance estimated by Houldcroft et al [11] using 677 samples from mixed continents. However, we observed that these estimates were highly affected by data transformation and the method to account for population structure. Repeating the GCTA analysis transforming the copy number measures in the same way that we did in our reported GWAS (*i*.*e*. population-wise inverse rank transformation), which rendered no increment of false positive associations; we obtained <5% and non-significant estimates of the proportion of genetic heritability. Correcting for population structure using the first 10 dimensions of the MDS alone, and using the Plink qassoc function, resulted in a large inflation factor in raw EBV copy number estimates (data not shown). This excess of false positives due to unaccounted structure was solved by transforming data using a population-wise inverse rank transformation. This suggests that previous estimates of variance >0.6 could have been inflated by uncorrected structure. On the other hand, our own non-significant and much lower estimate suggests that although the sample size in this study is the largest ever used for interrogating the genetic basis of EBV copy number variation among individuals, it is still lower for the recommended and reliable use of GCTA.

## Discussion

We report the largest GWAS study (n = 1753) ever performed to characterize the genetic basis of EBV copy number in LCLs, derived by EBV transformation of host B-cells. This work is based on the hypothesis that differences in EBV copy number in LCLs might offer an appropriate surrogate model to identify human genes implicated in the biology of the EBV infection of B-cells. Although, several lines of evidence support a strong link between in vivo EBV copy number and EBV associated malignancies [42–44], it is important to notice that EBV copy number measured in blood, plasma or serum might be unrelated to *in vitro* counts of EBV genomes per cell in EBV-transformed LCLs. Inter-individual variation in these two measures could reflect different processes. Our measure of EBV *in vitro* can be the consequence of several biological processes related to the immortalization process, such as the ability to entry and infect B-cells, the number of lytic reactivations or episomal establishment and B-cells transformation into LCLs. Our GWAS potentially highlighted host genes affecting those mechanisms and which constitute interesting candidates to follow up in the context of EBV related pathologies.

Two critical points to make our study possible were (i) obtaining a reliable estimation of relative viral copy number among individuals; and (ii) ensuring that relative EBV copy number in LCL is a stable phenotype that is maintained along different culture aliquots. *In vivo*, infected peripheral blood mononuclear cells (PBMCs) in healthy individuals are found in a proportion of 1–50 per 1,000,000, much lower than in LCLs cultures, and there exists variation between individuals. Importantly, the *in vivo* variation on this proportion within an individual measured over time, contributes only to 10% of the variance of the trait, and thus EBV copy number measured as the proportion of infected cells that can be considered a stable phenotype [45]. However, healthy individuals can show episodes of elevated viral load in PBMCs, possibly as a consequence of EBV reactivation [46]. As for the *in vitro* stability of EBV copy number very few published data are available. A recent study measured relative EBV copy number in LCLs during a yearlong experiment consisting in performing 6 cycles of freeze-thaw [47]. It is clear from this experiment that freeze/thaw has an effect on EBV copy number, particularly noticeable after the first cycle of newly transformed cells, when inter-individual variation gets confounded and intra-individual variation increases. For newly transformed cell lines, however, intra-individual variation is very low. Coriell Insitute stated upon enquire on a subset of 23 of our samples, that LCLs shipped to customers had been frozen/thawed not more than twice, with one sample that underwent 3 cycles.

In this study, we compared our *in silico* EBV copy number estimates from 13 LCLs with matched samples obtained from Coriell Institute by using TaqMan probe-based real-time PCR, which gave similar relative copy number. Thus, it validated our *in-silico* approach. Finally, we have shown here that cell culture passages do not cloak relative measures of EBV copy number at least in the 7 LCLs analyzed, where the proportion of variance explained by inter-individual differences was four-fold higher than the proportion explained by passages. All together, these observations support that measures in our study reflect stable inter-individual differences in viral copy number.

Our study confirmed inter-population variation in EBV copy number but not variation between the two genders. Although similar seroprevalence of EBV by sex is found in children and in early adolescence, higher antibody titers are found in females as observed in other viruses [48]. However, this observation can hardly be expected to replicate in LCLs in which EBV copy number is measured, rather than antibody titers, in a transformed B-cell culture produced in absence of a T-cell mediated immune response.

Our work has identified multiple genetic variants and genes associated with EBV copy number contained in 1,753 LCLs derived from 1KGP. Only *MACROD2* was tagged with a SNP surpassing the genome-wide P-value threshold. It is noteworthy that deletions in this gene has been related to gastric cancer, among other types of cancer, [49], a malignancy with strong bonds to EBV infection. In our region-based analysis, we have identified a number of potentially candidate genes, notably *KHDRBS2*, *FGD2*, *NRG3*, *DKK2*, *PIK3CB*, *CSMD1* and *CAND1*. These candidates are involved in biological process such as cell cycle control and transcription involving cell signaling pathways such as WNT, GPCRs, RHO GTPases and Interleukin receptor SHC signaling pathways. Many studies have shown that deregulation of these pathways are linked with EBV-associated malignancies such as NPC or lymphomas [50–54]. FGD2, for instance, activates CDC42 and has an important paralog, FGD4, which has been found to interact with EBV LMP1 protein to activate CDC42, a mechanism suggested to be implicated in the nasopharyngeal carcinoma tumurogenesis [26]. Candidate genes in Africans involve the Wnt signaling pathway, which has for long been suggested to be a pathway modulated by EBV infection [55,56]. While *DKK2* is a known modulator of the Wnt pathway [57], *LRRC61* is a putative target of miR-27a/27b. miR-27 miRNA are known activators of the Wnt signaling pathway [58].

One viral strategy for successful infection is the interference of the ubiquitin or ubiquitin-like systems to prime proteins for degradation. *BPLF1* is an EBV gene encoding a large tegument protein of the late phase of lytic infection, which possesses deubiquitinase activity. *BPLF1* is for instance responsible of the suppression of TLR-mediated activation of innate anti-viral immune system [59]. Also importantly, BPLF1 also acts on cullins interrupting the cullin-RING ligase (CRL) neddylation cycle, which in turn causes the accumulation of CRL substrates in the cell, producing an S-phase-like environment suitable for the EBV genome replication [60]. In order to interrupt the CRL cycle, BPLF1 also needs to inhibit the recruitment of CRL regulators, one of them being *CAND1* [33], which has been shown to be a potent inhibitor of EBV replication [34]. It is very remarkable that *CAND1* is one of the three candidate genes identified in Americans in association with EBV copy number.

We also observed that most GWAS signals turned out to be population-specific. Population differences in statistical power, though, could explain the apparent lack of shared associated loci. We reported EBV copy number-associated variants close to genes that deserve further study as they might play a role in EBV *in vivo* dynamics and ultimately in EBV-associated diseases. It is noteworthy, however, that independent analysis of populations at gene level using VEGAS2 rendered similar GO categories for Americans and Europeans. Those categories were related to cell adhesion, and this convergence was mainly due to the fact that low P-value SNPs in both populations, but particularly in Americans, were found near a genome cluster of proto-cadherines, that when tested for enrichment in Gene Ontologies, produced a significant enrichment in cell-adhesion categories. Cell-adhesion is a process that can be modulated by the EBV oncogenic protein LMP1 [61] and can be relevant for EBV cell-entry mechanism. For example, it is known that other cell-adhesion proteins, β1 integrin and α5 integrin, mediate attachment of EBV to oral epithelial cells [62,63].

Our measures of the proportion of genetic variance explaining EBV inter-individual are highly dependent on the transformation method (population-wise or mixing all populations) and affected by population structure. At least ~3000 individuals, almost twice our current sample size, are recommended as sample size for GCTA to obtain estimates of variance explained with a standard error <0.1 [64]. Therefore, estimates of genetic variance explained from this or previous studies such as [11] should be interpreted with caution.

The strength of this work is the establishment of several loci likely associated with EBV copy number, and thus potentially associated with EBV life cycle. Many of our suggested loci are actually close or within genes with a role in cell cycle control and cell signaling pathways and EBV-related cancers. The major drawback of this study lies in the relatively small LCL samples size to conduct a GWAS analysis. However, peaks identified in our GWAS show a desired decay of P-values with LD, which suggests that not a much larger sample size could start reducing present statistical uncertainties. This study sets the path for future experiments to uncover the molecular mechanism linking these genes with EBV copy number in LCLs.

## Supporting information

S1 Fig qq plot showing GWAS genome-wide p-values distribution in All Populations, Asian, African, American and European population subsets. (PDF)

S2 Fig Regional association plot produced by Locuszoom showing GWAS top SNP rs105452 from African population subset in purple and SNPs in the surrounding region colored depending on their degree of correlation (r2) with rs105452. Lower panel contains gene within this region. Solid blue lines represent recombination rates.
(PDF)

S1 Table List of a samples and populations derived from 1000 Genome Project with in silico estimated EBV copy number. (XLSX)

S2 Table ANOVA test output showing significant differences at the level of populations and continents. (XLSX)

S3 Table EBV copy number stability ANOVA test output. (XLSX)

S4 Table Continent wise list of GWAS top SNPs filtered by 10^−6^ and 10^−7^ p-value. (XLS)

S5 Table VEGAS2 output showing lists of genes ranked by p-value. (XLSX)
