# Correction: Genetic factors affecting EBV copy number in lymphoblastoid cell lines derived from the 1000 Genome Project samples

**DOI:** 10.1371/journal.pone.0196623

**Published:** 2018-04-30

**Authors:** Rajendra Mandage, Marco Telford, Juan Antonio Rodríguez, Xavier Farré, Hafid Laayouni, Urko M. Marigorta, Caitlin Cundiff, Jose Maria Heredia-Genestar, Arcadi Navarro, Gabriel Santpere

The fifth authors name is spelled incorrectly. The correct name is: Hafid Laayouni. The correct citation is: Mandage R, Telford M, Rodríguez JA, Farré X, Laayouni H, Marigorta UM, et al. (2017) Genetic factors affecting EBV copy number in lymphoblastoid cell lines derived from the 1000 Genome Project samples. PLoS ONE 12(6): e0179446. https://doi.org/10.1371/journal.pone.0179446
