# Supplementary figures and images for: Genetic factors affecting EBV copy number in lymphoblastoid cell lines derived from the 1000 Genome Project samples
Source: PLoS One. 2017 Jun 27;12(6):e0179446. doi: 10.1371/journal.pone.0179446 (PMC5487016; doi:10.1371/journal.pone.0179446)

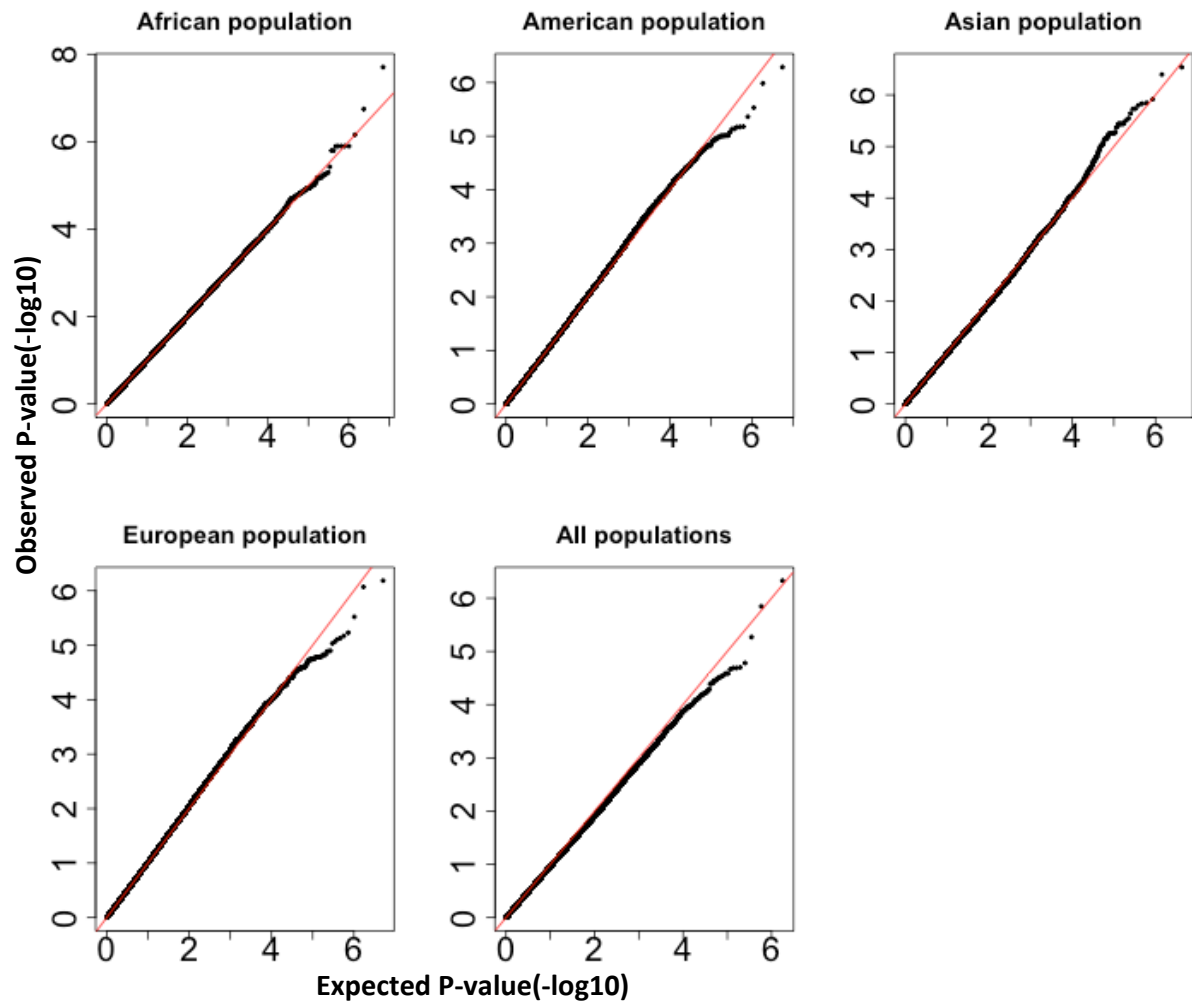

Supplement: S1 Fig — (PDF) [file pone.0179446.s001.pdf]

Plotted SNPs

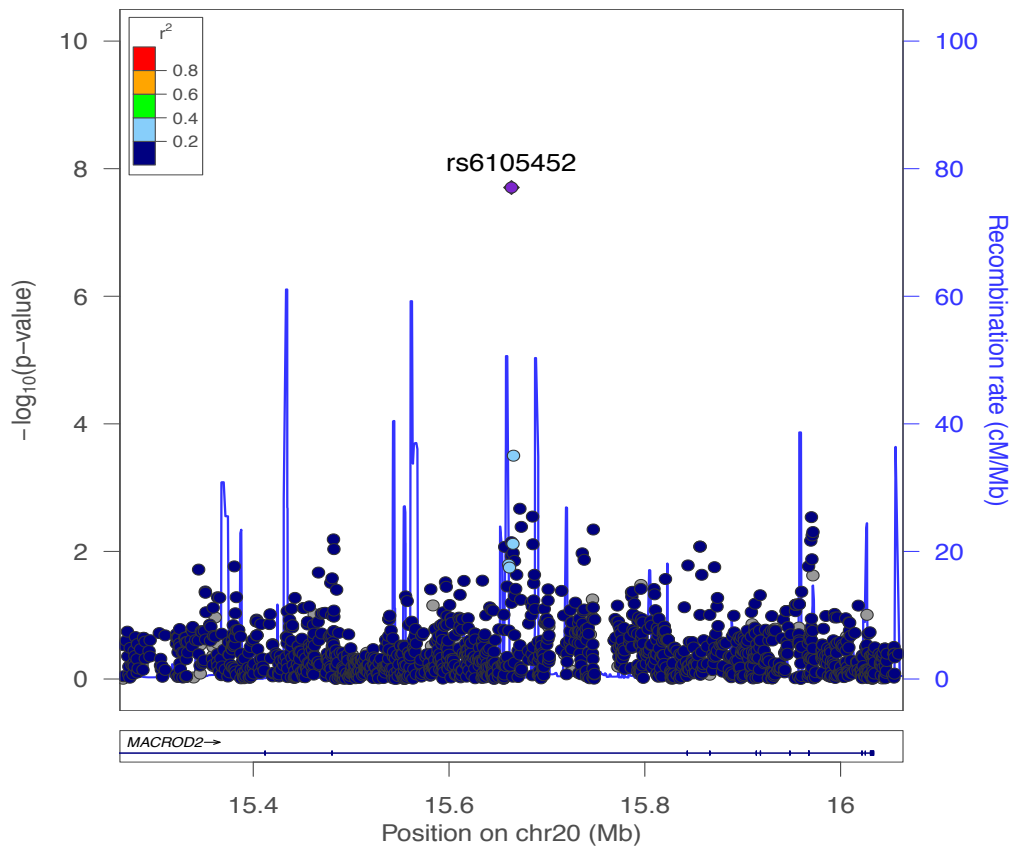

Supplement: S2 Fig — Lower panel contains gene within this region. Solid blue lines represent recombination rates. (PDF) [file pone.0179446.s002.pdf]
